# The histone demethylase Kdm3a is required for normal epithelial proliferation, ductal elongation and tumor growth in the mouse mammary gland

**DOI:** 10.18632/oncotarget.21380

**Published:** 2017-09-28

**Authors:** Li Qin, Yixiang Xu, Xiaobin Yu, Michael J. Toneff, Dabing Li, Lan Liao, Jarrod D. Martinez, Yi Li, Jianming Xu

**Affiliations:** ^1^ Department of Molecular and Cellular Biology and Dan L. Duncan Cancer Center, Baylor College of Medicine, Houston, TX, USA; ^2^ Institute of Biosciences and Technology, Texas A&M University Health Science Center, Houston, TX, USA; ^3^ Institute for Cancer Medicine and College of Basic Biomedical Sciences, Southwest Medical University, Sichuan, China

**Keywords:** Kdm3a, knockout mice, mammary gland growth, breast cancer, cyclin D1

## Abstract

Histone modification alters chromatin architecture to regulate gene transcription. KDM3A is a histone demethylase in the JmjC domain-containing protein family. It removes di- and mono- methyl residues from di- or mono-methylated lysine 9 of histone H3 (H3K9me2/me1). Recent studies have shown that Kdm3a plays an important role in self-renewal of embryonic stem cells, spermatogenesis, metabolism, sex determination and tumor angiogenesis. However, its role in mammary gland development and breast carcinogenesis remains unclear. In this study, we found that Kdm3a is expressed in the mouse mammary gland epithelial cells. Knockout of *Kdm3a* significantly increased H3K9me2/me1 levels in these epithelial cells, which correlated with markedly decreased mammary gland ductal elongation and branching in the intact knockout virgin mice. Furthermore, estrogen replacement in the ovariectomized Kdm3a knockout mice couldn’t rescue the retarded ductal growth. Moreover, transplantation of KO mammary gland pieces to wild type recipient mice showed slower ductal growth compared with that of WT gland pieces. Consistently, knockout of *Kdm3a* also reduced the proliferation rates and cyclin D1 expression in the mammary gland epithelial cells. In addition, *Kdm3a* knockout did not significantly change the latency of the polyoma middle T oncogene-induced mammary gland tumorigenesis. Tumor growth, however, was slowed which might be due to the decrease in cyclin D1 expression and tumor cell proliferation. We also found that Kdm3a binds and activates the cyclin D1 promoter. These results demonstrate that Kdm3a plays an important intrinsic role in promoting mammary gland ductal growth and tumor growth probably through enhancing cyclin D1 expression and cell proliferation.

## INTRODUCTION

Histone methylation status is reversible and counter-regulated by methyltransferases and demethylases. Histone demethylases remove methyl (CH3-) residues from methylated histone amino acids. The addition or removal of methyl marks alters chromatin accessibility at specific gene loci to regulate gene transcription [1]. Since the initial identification of the lysine-specific histone demethylase 1A (LSD1) [1], many demethylases have been reported [2]. According to their mechanisms, there are two main classes of histone demethylases, including a flavin adenine dinucleotide (FAD)-dependent amine oxidase and an Fe(II) and α-ketoglutarate-dependent dioxygenase [3]. These demethylases contain SWIRM1 (Swi3, Rsc, and Moira domain), Jumonji (N/C terminal domains), PHD-finger, zinc-finger and amine oxidase domains for recognizing the methylated amino acid substrate, catalyzing the demethylation reaction and interacting with other cofactors [4]. The JmjC-domain-containing proteins remove the methyl groups from histones through a hydroxylation reaction that requires alpha-ketoglutarate and Fe(II) as cofactors [5]. KDM3A (JMJD1A, JHDM2A or JHMD2A) contains a jmjC domain, a zinc finger, and a LXXLL motif for interacting with steroid hormone receptors [6, 7]. KDM3A activates gene transcription by demethylating the di- and mono-methylated lysine 9 (K9) of histone H3 (H3K9me1/me2) [3]. Studies using knockout (KO) mice have revealed important roles of Kdm3a in metabolic homeostasis, male sex organ development and male reproductive function [5, 7-12]. For example, KO of *Kdm3a* in mice decreased fat oxidation and glycerol release from brown fat and skeletal muscle, leading to an adult onset-obesity phenotype [10, 13]. KO of *Kdm3a* affected the expression of mammalian Y chromosome sex-determining gene Sry and caused some KO mice to undergo male-to-female sex reversal [12]. KO of *Kdm3a* in mice also disturbed the expression of the Crem coactivator Act and the recruitment of cAMP-responsive element modulator (Crem) to chromatin, resulting in the decreased expression of Tnp1/2 (transition proteins 1 and 2) and Prm1/2 (protamines 1 and 2). Since all of these proteins are required for chromatin condensation in the spermatids, *Kdm3a* KO male mice displayed a severe oligozoospermia and infertility [5, 8]. However, the function of Kdm3a in mammary gland development and morphogenesis are still unknown.

Accumulated evidence suggests that Kdm3a may be implicated in the development and progression of multiple malignancies. In human breast cancer cells, knockdown or inactivation of KDM3A abolished the recruitment of estrogen receptor alpha (ERα) to its target gene enhancer/promoter regions, leading to a downregulation of ERα target genes and attenuated response to estrogen-stimulated cell growth [14]. KDM3A was found up-regulated in the gastric cancer tissues and cell lines. The elevated KDM3A expression positively correlates with the invasion depth and lymph node metastasis, suggesting that Kdm3a may be an independent prognostic predictor of overall survival [15]. In human prostate tumors, KDM3A expression positively correlates with c-Myc expression. In prostate cancer cell lines, KDM3A promotes androgen receptor activity that in turn upregulates c-Myc expression and also inhibits the E3 ubiquitin ligase HUWE1 which prevents c-Myc protein degradation. These findings suggest that Kdm3a may promote prostate cancer cell proliferation and survival by upregulating c-Myc expression [16]. However, some studies also considered KDM3A as a possible tumor suppressor. For example, the hypoxia-induced KDM3A expression was found to be markedly reduced in human germ cell-derived tumors including seminomas, yolk sac tumors, and embryonal carcinomas. Loss of *Kdm3a* function increased the growth of human germ cell tumors. Furthermore, the H3K9 methyltransferase G9a could be induced by hypoxia to increase H3K9 methylation, suppress the expression of anti-angiogenesis factors such as Robo4, Igfbp4, Notch4, and Tfpi and hence promote angiogenesis and tumor growth. However, the presence of KDM3A could oppose the angiogenic role of G9a to inhibit angiogenesis and tumor growth [17]. Despite these studies, the role of Kdm3a in breast tumorigenesis has not been defined in any genetically engineered mouse models.

In this study, we found that Kdm3a is expressed in the mouse mammary epithelial cells. KO of the *Kdm3a* gene in female mice decreased the epithelial proliferation and ductal elongation of their mammary glands. Furthermore, KO of *Kdm3a* also significantly slows down the polyoma middle T (PyMT) oncogene-induced mammary tumor growth by reducing cyclin D1 expression and cell proliferation. Our findings suggest that Kdm3a plays an important genetic role to promote mammary gland epithelial proliferation, ductal growth and tumor growth *in vivo*.

## RESULTS

### Kdm3a protein is differentially expressed in the luminal epithelial cells of mouse mammary glands at different ages

**Figure 1:**
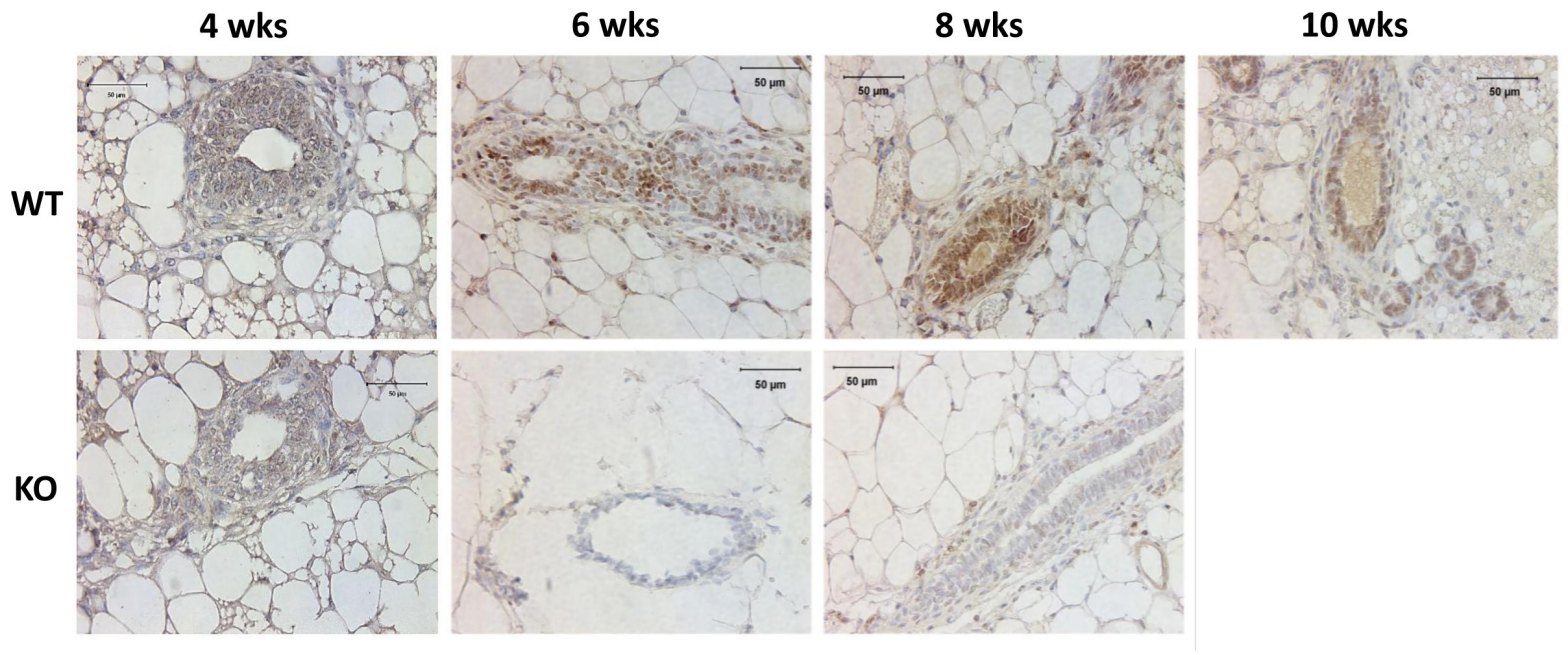
Analysis of Kdm3a protein in the mouse mammary gland by IHC Mammary gland tissues were isolated from WT and Kdm3a KO female mice with the indicated ages in weeks (wks). Paraffin-embedded tissue sections were subjected to IHC for Kdm3a (brown color). Tissues on slides were counter-stained with hematoxylin. Kdm3a KO mammary gland tissues served as the negative control of Kdm3a staining. Scale bars represent 50 µm in length.

To determine the expression of Kdm3a in the mammary gland, we performed IHC on paraffin sections of mammary glands collected from *Kdm3a* KO and WT virgin mice. We found positive Kdm3a signals are mainly distributed in luminal epithelial cells of WT mammary glands. In 4-week-old mammary glands, Kdm3a signal intensity in epithelial cells was weak, indicating the expression of Kdm3a is low at that time point. In the 6 week-old mammary glands the signal intensity in epithelial cells was drastically increased and peaked at 8 weeks. However the signal declined at 10 weeks of age (Figure 1). Fat cells, few myoepithelial cells and immune cells also exhibited positive staining. As expected, there is no obvious signal observed in *Kdm3a* KO mammary glands at all examined time points. These results indicated that Kdm3a is expressed in mammary gland luminal epithelial cells and its expression displays dynamic changes with a peak at 8 weeks, which correlates with rapid ductal growth at that stage of development.

### Knockout of *Kdm3a* increases H3K9me1 and H3K9me2 in the mammary epithelial cells

**Figure 2:**
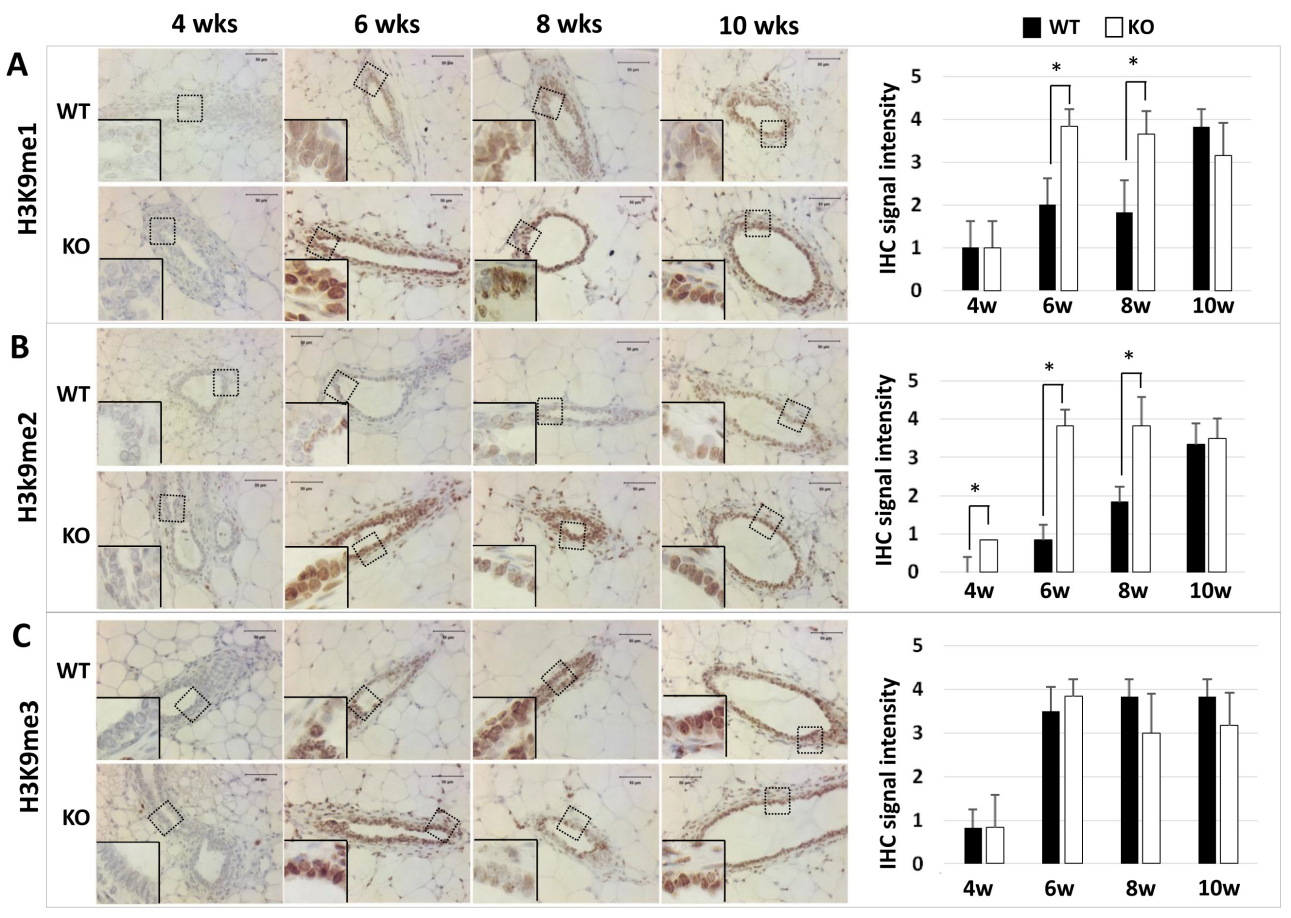
Detection of H3K9me1 (Panel A), H3K9me2 (Panel B), and H3K9me3 (Panel C) by IHC in the mammary glands of WT and *Kdm3a* KO mice at the indicated ages in weeks (wks) IHC (brown color) was performed using Kdm3a antibody with de-paraffinized mammary gland tissue sections prepared from 5 mice in each age and genotype group. Tissue slides were counter-stained with hematoxylin and imaged under microscope at the magnification of 200×. IHC-staining intensity was scored in a scale ranged from 0 to 4. The bar graphs present the average scores of 5 samples in each group. *, *p* < 0.05 by unpaired Student’s *t* test.

Kdm3a can specifically remove mono- or di-methyl residues from H3K9me1 or H3K9me2 to regulate gene transcription [5, 8, 10]. To determine the effects of *Kdm3a* KO on the status of H3K9 methylation, we performed immunohistochemistry (IHC) to compare the levels of H3K9me1, H3K9me2 and H3K9me3 in the mammary gland epithelial cells of WT and *Kdm3a* KO mice. We found that H3K9me1, H3K9me2 and H3K9me3 immunoreactivities were very low or undetectable in the mammary gland epithelial cells of 4-week-old WT and *Kdm3a* KO mice. In the mammary glands of 6-, 8- and 10-week-old mice, we found different intensities of H3K9me1 and H3K9me2 immunoreactivities in the nuclei of WT and *Kdm3a* KO mammary gland epithelial cells (Figure 2). Specifically, at the ages of 6 and 8 weeks, H3K9me1 and H3K9me2 only slightly increased in the mammary epithelial cells of WT mice, but both dramatically increased in the mammary epithelial cells of *Kdm3a* KO mice. At the age of 10 weeks, the levels of H3K9me1 and H3K9me2 remained the same in the epithelial cells of *Kdm3a* KO mammary glands. However, both H3K9me1 and H3K9me2 significantly increased in the epithelial cells of WT mammary glands, which matched their levels to those in the *Kdm3a* KO epithelial cells at this age (Figure 2A and 2B). However, H3K9me3 levels were comparable between WT and *Kdm3a* KO mammary glands at ages of 6, 8 and 10 weeks (Figure 2C). These results demonstrate that Kdm3a deficiency significantly increases H3K9me1 and H3K9me2 levels in the mammary gland epithelial cells of 6- and 8-week-old mice, which matches the stages of fast mammary ductal elongation.

### Knockout of *Kdm3a* results in retarded mammary gland ductal growth

**Figure 3:**
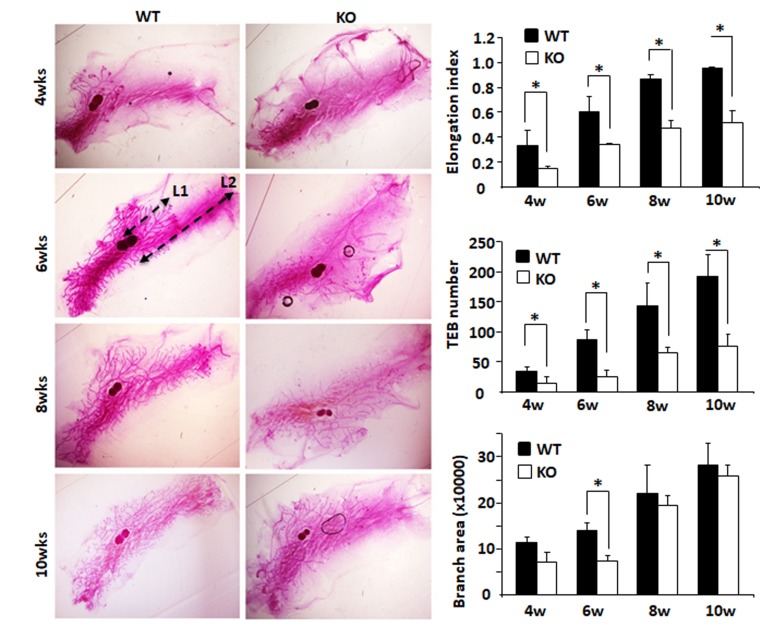
Mammary gland ductal morphogenesis in WT and *Kdm3a* KO virgin mice Mammary glands were isolated from female *Kdm3a* KO and WT mice with the indicated ages in weeks (wks) and stained with carmine alum. Five mice were analyzed for each genotype and age group. Mammary gland ductal elongation index was the ratio of L1/L2 as illustrated for the WT/6w mammary gland. L1 is the distance from the lymph node to the leading edge of the mammary ductal growth. L2 is the distance from the lymph node to the distal edge of the mammary fat pad. The number of terminal-end buds (TEBs) were counted for each gland and the average numbers in five glands for each group were presented. Branch distribution areas (pixels) were measured on the electronic images by using the NIH ImageJ software. *, *p* < 0.05 by unpaired Student’s *t* test.

Mouse mammary gland ductal growth occurs postnatally beginning with the commencement of puberty. At about 3 weeks of age, the terminal end buds (TEBs) appear with the secretion of ovarian hormones and the mammary gland ductal elongation starts [18]. In the 4-week-old WT mice, abundant ductal branches were observed in the mammary glands, and their TEBs had extended beyond the lymph nodes. However, in the 4-week-old *Kdm3a* KO mice, there were significantly fewer mammary gland ductal branches. The TEBs of these branches had not yet reached the lymph nodes in most of these mice. The TEB number and branch area in *Kdm3a* KO mammary glands were much less and smaller than that in WT glands and significant differences of ductal elongation and number of TEBs were detected at 4 weeks (Figure 3). At 6 weeks of age, mammary gland branches in *Kdm3a* KO mice were still rare compared with that of WT mice at the same age. Furthermore, the leading edge of TEBs just reached the lymph nodes, which was significantly slower than the ductal elongation in WT mammary glands at the same age. Consistently, the average ductal branch area in *Kdm3a* KO mammary glands was significantly smaller compared with the average ductal branch area of WT mammary glands (Figure 3). At 8 and 10 weeks of age, the ductal elongation in WT mammary glands had almost or fully reached the distal ends of the fat pads; the number of their TEBs remained high and their ductal branches had occupied the entire fat pads. However, in the *Kdm3a* KO mammary glands, the ductal elongation was still significantly retarded and the number of TEBs remained much fewer compared with that of age-matched WT mammary glands (Figure 3). These results suggest that Kdm3a is required for normal growth of the mammary gland ducts.

### *Kdm3a* KO-caused mammary gland ductal growth retardation cannot be rescued by estrogen replacement

**Figure 4:**
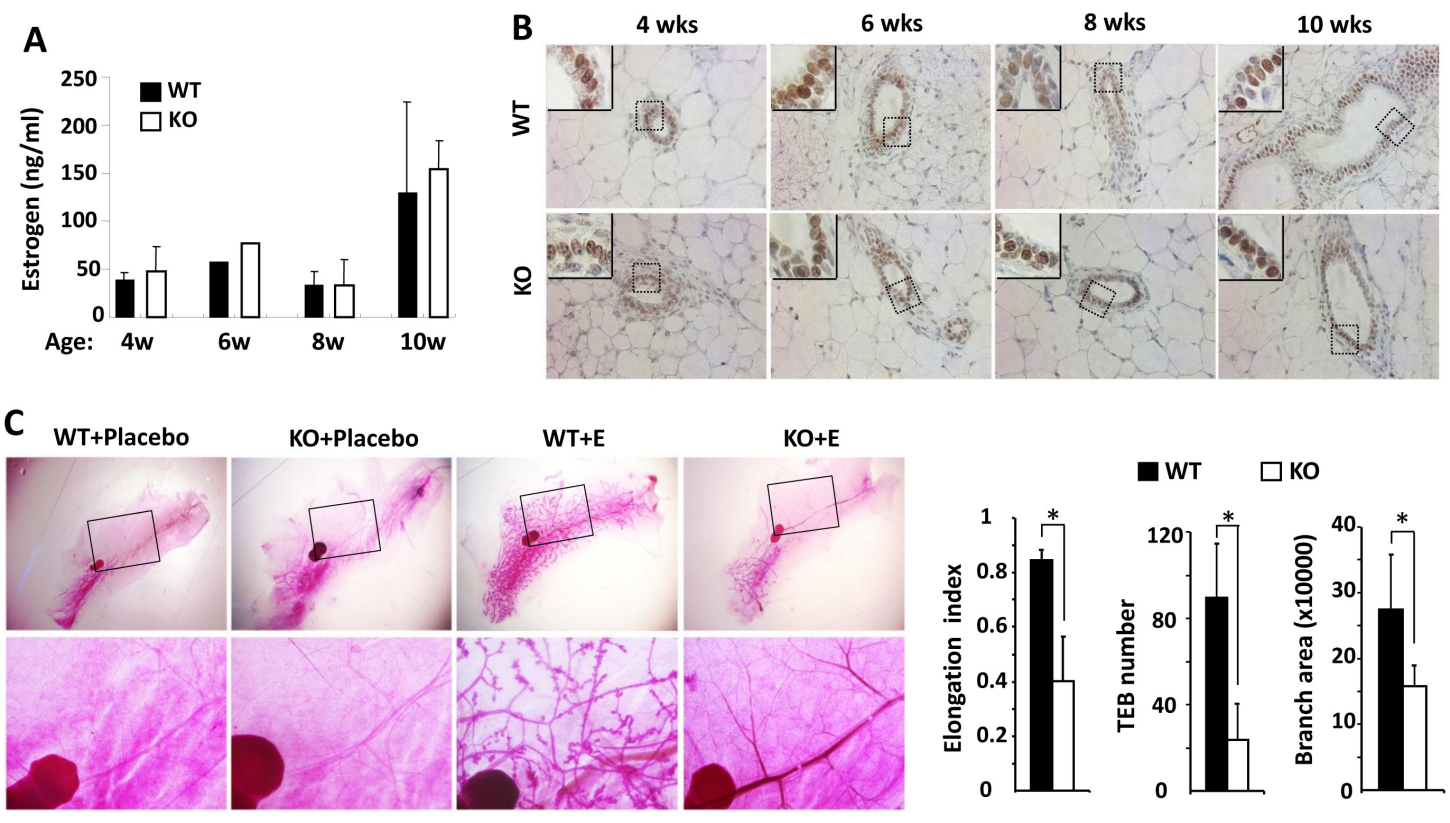
Estrogen replacement does not rescue the retarded mammary gland ductal growth in the female *Kdm3a* KO mice **A.** Estrogen concentrations in the serum samples of female WT and Kdm3a KO mice (*n* = 5 in each group) with the ages indicated in weeks (wks). No significant difference was found. **B.** ERα IHC in the mammary gland sections of female WT and Kdm3a KO mice with the ages indicated in weeks (wks). **C.** Carmine alum-stained mammary glands of ovariectomized WT and *Kdm3a* KO mice treated with placebo or estrogen pellet (E) (*n* = 5 in each treatment group) as indicated. The elongation index, TEB number and branch area of the mammary glands in estrogen-treated WT and *Kdm3a* KO mice were quantitively assayed and statistically compared. *, *p* < 0.05 by unpaired Student’s *t* test.

Estrogen plays a principal role in mammary gland ductal growth. To determine if estrogen levels in *Kdm3a* KO and WT mice could account for the differences in mammary gland ductal growth, we measured 17β-estradiol concentration in serum samples prepared from WT and *Kdm3a* KO female mice at 4, 6, 8 and 10 weeks of age. To our surprise, we didn’t find any discrepancy of 17β-estradiol levels in the serum samples of *Kdm3a* KO mice *versus* WT mice at all examined time points (Figure 4A). Furthermore, we also compared ERα expression by IHC in the mammary tissues collected from WT and *Kdm3a* KO mice at matched ages. We found no difference in either the immunostaining signal intensity or the subcellular localization of ERα in the epithelial cells of *Kdm3a* KO mammary glands *versus* WT mammary glands at all ages examined (Figure 4B). Next, we further examined whether estrogen replacement could rescue the retarded mammary gland growth in *Kdm3a* KO mice. We ovariectomized 4-week-old WT and *Kdm3a* KO mice and then treated these mice with placebo or estrogen pellets for 2 weeks. As expected, placebo treatment did not induce any ductal growth in all mice. Estrogen-treated WT mice showed robust mammary gland ductal growth; while estrogen-treated *Kdm3a* KO mice exhibited a significantly slower growth of their mammary gland ducts *versus* that in WT mice (Figure 4C). Quantitative analysis revealed that the ductal elongation index, TEB number and branch area of the estrogen-treated *Kdm3a* KO mammary glands were only 44%, 32% and 60% of that of the estrogen-treated WT mammary glands, respectively (Figure 4C). We also found that the mammary gland ductal growth and side branches in the ovariectomized *Kdm3a* KO mice treated with both estrogen and progesterone were still significantly slower and lesser compared with that in WT mice receiving the same treatment (data not shown). These results indicate that Kdm3a is not required for estrogen-induced ductal growth in the mammary gland.

### The role of Kdm3a in promoting mammary gland ductal growth is epithelial-cell-intrinsic

**Figure 5:**
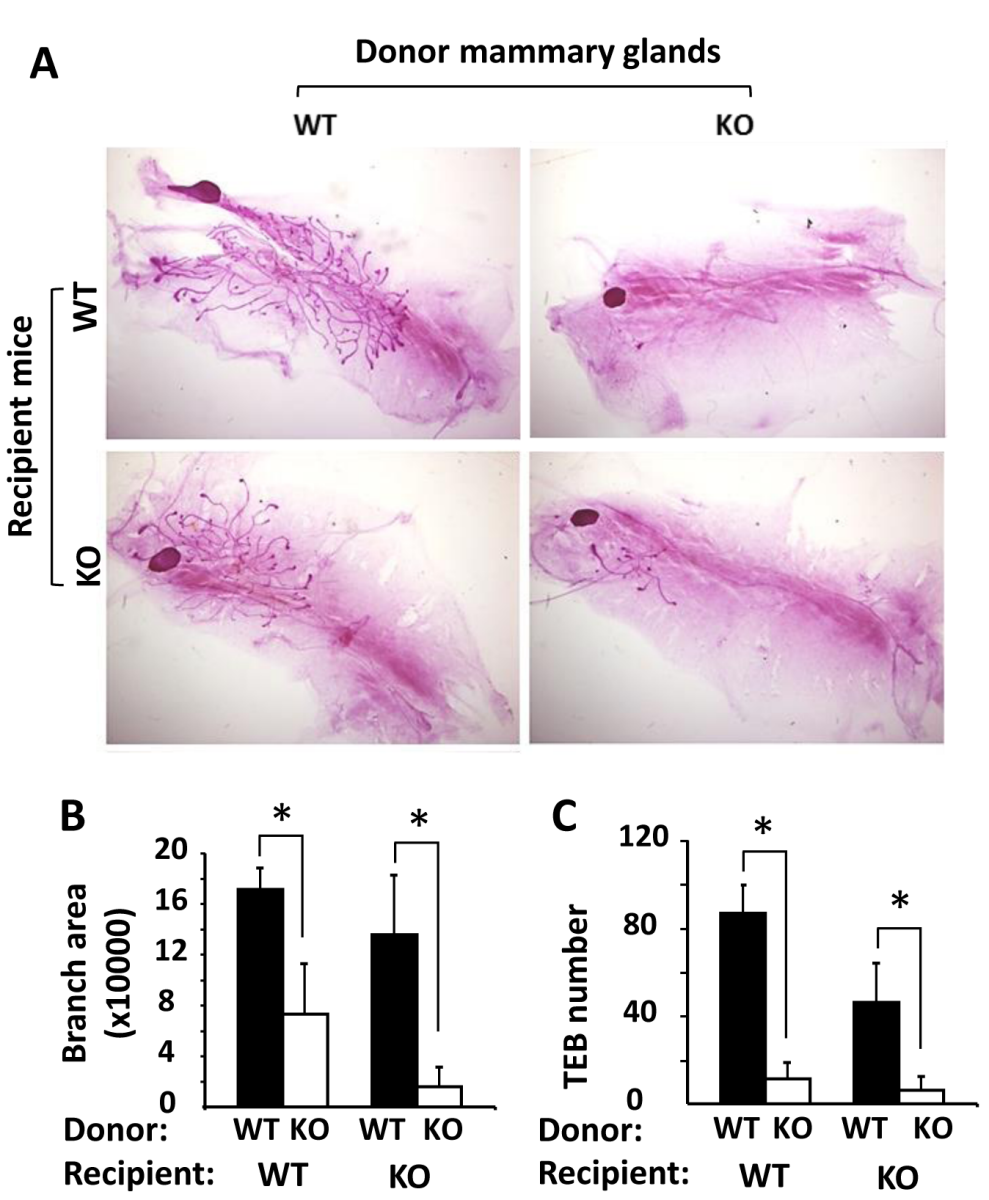
Mammary gland transplantation assay **A.** Representative images of carmine alum-stained mammary glands in the recipient WT or *Kdm3a* KO mice (*n* = 5 in each group). These glands were re-generated from implanted donor WT and *Kdm3a* KO mammary gland tissues as indicated. **B.** & **C.** The mammary gland ductal branch areas and TEB numbers of the indicated mammary glands were quantitatively assayed and statistically compared by unpaired Student’s t test. *, *P* < 0.05.

To determine whether the retarded mammary gland ductal growth in *Kdm3a* KO mice was caused by Kdm3a deficiency in the mammary gland epithelial cells or by the changes of systemic factors and/or local tissue environment, we performed reciprocal mammary gland transplantation assays. After WT mammary gland tissues were transplanted into the cleared mammary gland fat pads of WT or *Kdm3a* KO mice for 4 weeks, robust outgrowth of mammary ducts were observed in both groups of recipient mice. However, after *Kdm3a* KO mammary gland tissues were transplanted into the cleared mammary gland fat pads of *Kdm3a* KO recipient mice, very little mammary gland ductal outgrowth could be observed (Figure 5A). In the mammary gland fat pads of either WT or *Kdm3a* KO recipient mice, the areas occupied by the mammary gland ductal branches and the number of TEBs derived from WT mammary gland tissues were significantly bigger and more than that derived from *Kdm3a* KO mammary gland tissues (Figure 5B and 5C). We also noticed that the ductal outgrowth from WT mammary tissues in WT recipients was slightly faster than that in *Kdm3a* KO recipients. Furthermore, the ductal outgrowth from *Kdm3a* KO mammary tissues in WT recipients was a little faster than that in *Kdm3a* KO recipients (Figure 5A-5C). These results indicate that Kdm3a expressed in the mammary gland epithelial cells plays a major intrinsic role, while Kdm3a expressed in other cells plays a minor role in supporting the mammary gland ductal growth.

### Kdm3a is required for normal proliferation and cyclin D1 expression in the mammary gland epithelial cells during the pubertal fast growing phase of mammary gland development

**Figure 6:**
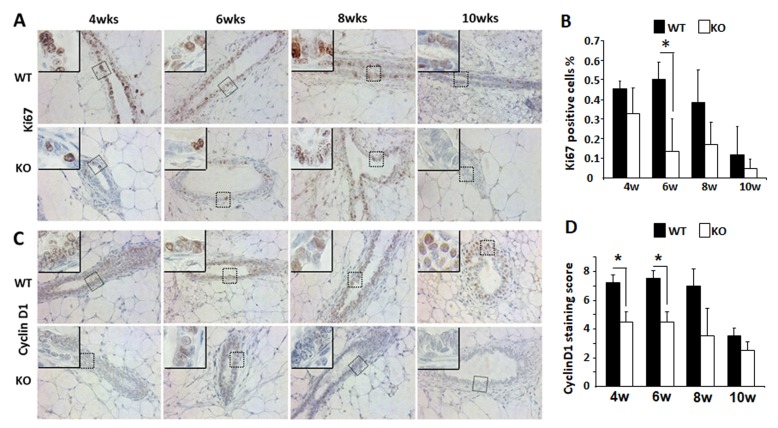
Analysis of the epithelial cell proliferation and cyclin D1 expression by IHC in the WT and *Kdm3a* KO mammary glands **A.** Detection of Ki67-expressing epithelial cells by IHC in the mammary glands of WT and *Kdm3a* KO mice with the indicated ages in weeks (wks). For each genotype, 5 mammary glands (one per mouse) were analyzed in each age group. Representative images are presented. **B.** Percent of Ki67-positive epithelial cells to total epithelial cells in the WT and *Kdm3a* KO mammary glands in mice with the indicated ages in weeks (w). Five images were taken from different areas of each immunostained mammary gland tissue section. For each genotype group, 5 mammary glands from 5 individual mice were analyzed. The number of Ki67-positive and total mammary gland epithelial cells were respectively counted. The data are presented as mean ± standard deviation (SD). **C.** IHC for cyclin D1 performed with the same groups of mammary gland sections for Ki67 IHC (Panel A). **D.** Immunoreactive scores of cyclin D1 in the WT and *Kdm3a* KO mammary glands of the same mouse groups used for analyzing Ki67-positive cells (Panel B). * in Panels B and D, *p* < 0.05 by unpaired *t* test.

To understand how *Kdm3a* KO caused retarded mammary gland ductal growth at the cellular level, we evaluated cell proliferation by immunostaining Ki67, a commonly used marker for proliferating cells (Figure 6A). We found that the percent of Ki67 positive epithelial cells was 45% at 4 weeks of age and increased to 51% at 6 weeks of age in WT mammary glands. Then the percentage decreased to 40% at 8 weeks of age and further dropped to 20% at 10 weeks of age in WT mammary glands. However, the percent of Ki67 positive epithelial cells were lower in *Kdm3a* KO mammary glands, which were 34%, 15%, 17% and 5% at 4, 6, 8 and 10 weeks of age, respectively (Figure 6B). Interestingly, in the fast growing stage of mammary gland ducts at 6 weeks of the age, the percent of Ki67-positive epithelial cells in WT mammary glands was 3.4 fold higher than that in *Kdm3a* KO mammary glands (Figure 6B). These results indicate that Kdm3a is required for maintaining the fast epithelial proliferation in the mammary gland during puberty, which is consistent with the retarded mammary gland ductal growth observed in *Kdm3a* KO mice.

Growth factor and estrogen signaling pathways induce cyclin D1 expression and in turn, cyclin D1 accelerates G1 phase progression of the cell cycle [19]. Since the cellular proliferation rate was found decreased in *Kdm3a* KO mammary gland epithelial cells, we next compared cyclin D1 protein expression in the mammary gland epithelial cells of WT and *Kdm3a* KO mice by IHC (Figure 6C). Immunostaining revealed that the cyclin D1-positive epithelial cells in *Kdm3a* KO mammary glands were 30-35% of total epithelial cells from 4 to 8 weeks of age and dropped to 10% at 10 weeks of age. Moreover in WT mammary glands the cyclin D1-positive epithelial cells were approximately 50-60% of the total epithelial cells at 4 to 8 weeks of age and 20% at10 weeks of age. The intensity of cyclin D1 immunoreactivity was also higher in WT mammary gland epithelial cells *versus Kdm3a* KO mammary gland epithelial cells in most samples examined. Quantitative analysis of the combined immunostaining scores demonstrated that the overall cyclin D1 expression was significantly lower in *Kdm3a* KO *versus* WT mammary gland epithelial cells at 4 and 6 weeks of age (Figure 6D). These results suggest that Kdm3a is required for normal cyclin D1 expression during the pubertal fast mammary gland ductal growth phase at 4-6 weeks of age.

### *Kdm3a* KO showed no obvious effect on mammary gland tumor initiation but significantly slowed down tumor growth

**Figure 7:**
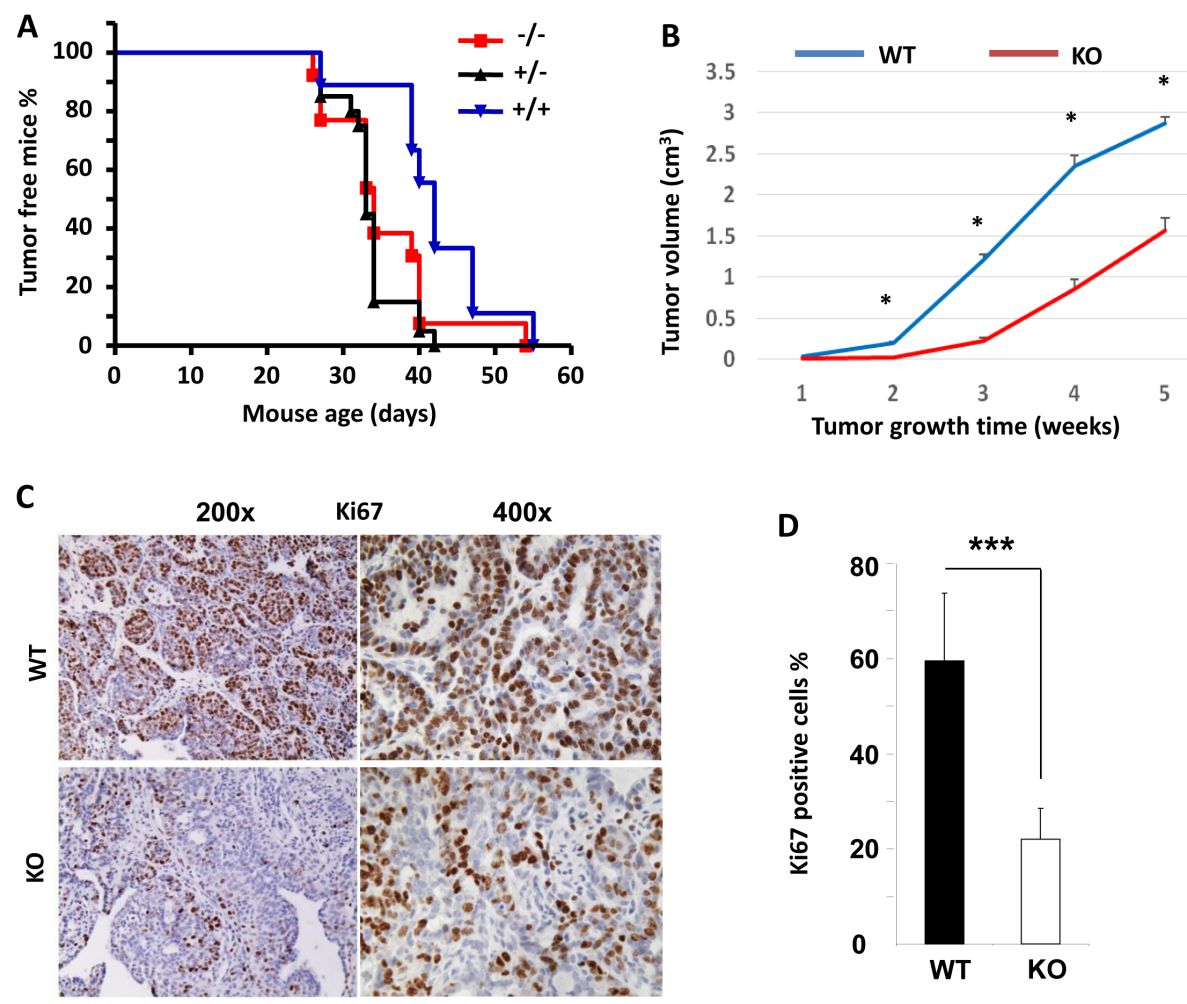
The effects of *Kdm3a* KO on the initiation and growth of PyMT-induced mammary gland tumors and the expression levels of Ki67 in the tumor cells **A.** The initiation curves of palpable mammary gland tumors induced by intraductal injection of RCAS-PyMT virus into the mammary glands of MMTV-TVA×WT (+/+), MMTV-TVA×*Kdm3a*^+/-^ (+/-) and MMTV-TVA×*Kdm3a*^-/-^ (-/-) mice (*n* = 12 in each group). No significant differences were detected among the three groups by Log-rank test (*p* > 0.05). **B.** The growth curves of mammary tumors induced by RCAS-PyMT virus in MMTV-TVA×WT (WT) and MMTV-TVA×*Kdm3a*^-/-^ (KO) mice (*n* = 12 in each group). *, *p* < 0.05 by unpaired *t* test. **C.** IHC for Ki67 protein in the mammary tumors of MMTV-TVA×WT (WT) and MMTV-TVA×*Kdm3a*^-/-^ (KO) mice (*n* = 12 in each group) shown in Panel B. Images were taken at 200x and 400x magnifications. **D.** Percent of Ki67-positive tumor cells to total cells. Data are presented as mean ± SD, *n* = 12. ***, *p* < 0.001 by unpaired Student’s *t* test.

Since *Kdm3a* is required for mammary gland epithelial cell proliferation and cyclin D1 expression, we examined its role in mammary tumorigenesis in a genetically engineered mouse model. We generated *Kdm3a*^+/+^XMMTV-TVA (*Kdm3a* WT with the avian subgroup A receptor gene, TVA, thereafter designated as TVA), *Kdm3a*^+/-^xTVA (*Kdm3a* heterozygous with TVA), and *Kdm3a*^-/-^xTVA (*Kdm3a* KO with TVA) mice. Then, we introduced RCAS-PyMT (polyoma middle T antigen) avian virus into the mammary gland ducts of these mice to infect the TVA-expressing mammary gland epithelial cells for induction of mammary gland tumors. Unexpectedly, the latency time before palpable mammary tumor formation showed no significant difference among the three groups of mice, indicating that Kdm3a is not essential for the development of PyMT expression-induced mammary tumorigenesis. However, the mammary gland tumors in *Kdm3a*^-/-^xTVA mice grew much slower than that in TVA control mice after the palpable tumors were detected (Figure 7B). Analysis of Ki67 expression by IHC revealed that Ki67-positive cell number was dramatically reduced in *Kdm3a* KO tumors *versus* WT tumors (Figure 7C and 7D). These results indicate that Kdm3a is required for supporting mammary tumor growth, which is consistent with its role to increase tumor cell proliferation.

### Kdm3a plays a direct role in upregulating cyclin D1 expression

**Figure 8:**
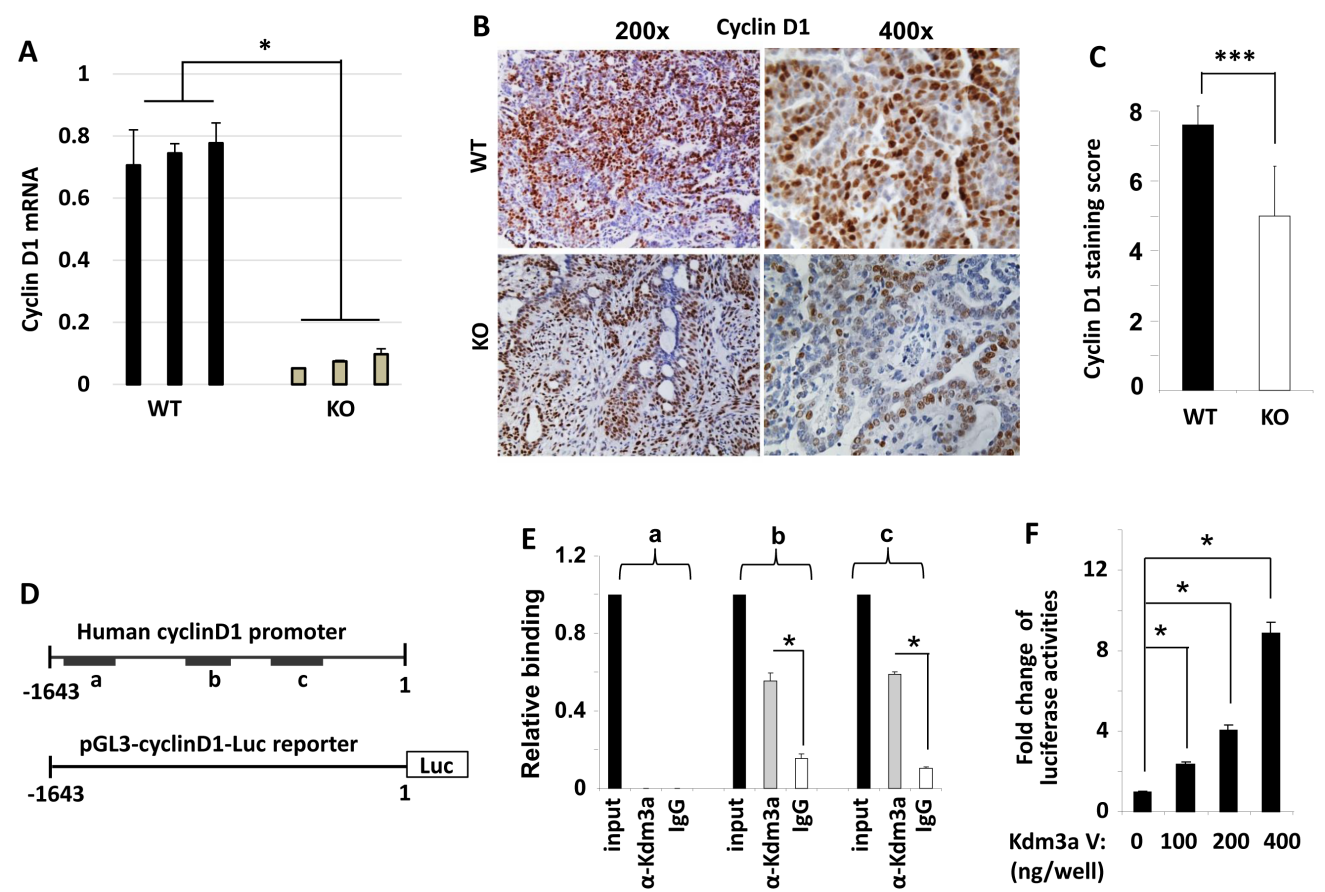
Kdm3a directly regulates cyclinD1 expression **A.** QPCR measurement of cyclin D1 mRNA in the RCAS-PyMT-induced mammary tumors in WT and *Kdm3a* KO mice (*n* = 3). **B.** Representative images of cyclin D1 IHC (brown color) in WT and *Kdm3a* KO mouse mammary tumors. **C.** The immunoreactivity of cyclin D1 in WT and *Kdm3a* KO mammary tumors (*n* = 12) was scored using the Allred score system and the average score of each genotype group was presented as mean ± SD. **D.** The 5’ non-coding regulatory sequence (region -1780 to 1 bp) of the human cyclin D1 gene. Regions a, b and c that are amplified by QPCR in ChIP assay are indicated with black bars. The cyclin D1 promoter-luciferase reporter construct contains the 5’ non-coding region from -1643 to 1 bp in the pGL3 plasmid. **E.** ChIP assay was performed with Kdm3a antibody and DNA-protein complex extracted from MCF-7 cells. The eluted DNA was used as template for QPCR analysis using specific primers for amplifying regions a, b or c. Immunoprecipitation with same amount of non-immune IgG served as a background control, and 5% of the DNA-protein complex material was assayed by QPCR as an input control. Data were normalized to input control and presented as mean ± SD. **F.** HeLa cells were transfected with the pGL3-cyclin D1 promoter-Luc reporter DNA (shown in panel D) and the different amounts of Kdm3a expression vector DNA as indicated. Luciferase activity was assayed 48 hours post the transfection, and normalized to total cellular protein amount assayed for each sample. Results were presented as mean ± SD. ** or *** in relevant panels, *p* < 0.01 or *p* < 0.001 by unpaired Student’s *t* test or One-Way ANOVA.

Cyclin D1 is a key regulator of cell cycle and its expression is regulated by extracellular cell growth stimuli such as growth factors and hormones. To investigate the role of Kdm3a in modifying cyclin D1 expression in the mammary tumor cells, we measured cyclin D1 mRNA and protein expression by real-time polymerase chain reaction (QPCR) and IHC. We found that cyclin D1 mRNA was drastically decreased in Kdm3a KO mouse mammary gland tumors *versus* WT tumors (Figure 8A). Both the immunostained positive cell number and the immunostaining intensity were reduced in Kdm3a KO tumors *versus* WT tumors. Semi-quantitative analysis of tumors from multiple mice confirmed that the average immunoreactive score of cyclin D1 in Kdm3a KO tumors was significantly lower than that in WT tumors (Figure 8B and 8C). These results indicate that Kdm3a plays a role in upregulating cyclin D1 expression in the PyMT-induced mouse mammary gland tumors. Consistantly, ectopic expression of KDM3A in MCF-7 cells increased cyclin D1 mRNA levels. On the contrary, knockdown of KDM3A in MCF-7 cells reduced cyclinD1 mRNA (Data not shown).

To assess whether Kdm3a could directly promote cyclin D1 expression at the transcriptional level, we performed ChIP assay to examine if Kdm3a was recruited to the 5’ regulator sequence of the cyclin D1 gene containing the cyclin D1 promoter in MCF-7 human breast cancer cells. In this assay, a Kdm3a antibody was used to immunoprecipitate Kdm3a and its respective DNA complexes; after which QPCR was performed to measure 3 short DNA regions (termed as regions a, b and c in Figure 8D) located in the 5’ non-coding regulatory sequence of the human cyclin D1 gene. In this assay, a small portion of the input DNA-protein complex material and the non-immune IgG-precipitated materials were used as positive and background control samples, respectively. Our assay revealed that Kdm3a was associated with regions b (-1318 to -1140 bp) and c (-948 to -687 bp), but was not associated with region a (-1879 to -1671) (Figure 8D and 8E). These results suggest that Kdm3a may play a direct role in upregulating cyclin D1 transcription.

To further define the direct effect of Kdm3a on the transcriptional activity of the cyclin D1 promoter, we generated a promoter-luciferase reporter construct by cloning a 5’ DNA fragment (-1643 to 1 bp) of the cyclin D1 gene to the upstream of the luciferase sequence. This DNA fragment contained both Kdm3a-binding regions b and c (Figure 8D). When HeLa cells were co-transfected with the same amount of the cyclin D1 promoter-reporter plasmid and different amounts of Kdm3a expression plasmids, we found that Kdm3a robustly increased the luciferase reporter activity in a dose-dependent manner (Figure 8F). In addition, we also overexpressed Kdm3a in MCF-7 cells and observed Kdm3a-enhanced cyclin D1 expression (Data not shown). Taken together, these results demonstrate that Kdm3a directly associates with the cyclin D1 promoter to promote cyclin D1 expression.

## DISCUSSION

Kdm3a has been shown to play important roles in spermatogenesis, metabolism, sex determination, stem cell self-renewal and differentiation [5, 8-10, 12]. In this study, we systematically analyzed the expression and the role of Kdm3a in mammary gland morphogenesis. We demonstrated that Kdm3a is differentially expressed in the mouse mammary gland epithelial cells at different ages, and high expression levels are correlated with the rapid mammary gland ductal growth during puberty. This expression pattern suggests that Kdm3a may play a role in promoting mammary gland ductal growth. Indeed, KO of *Kdm3a* reduced the TEB number and retarded mammary gland ductal elongation in virgin mice. In agreement with the proposed role of Kdm3a in supporting mammary gland ductal growth, we also found that KO of *Kdm3a* significantly decreased the proliferation rate of the mammary epithelial cells during the fast growing stage of mammary glands during puberty. Interestingly, the retarded mammary gland ductal growth in *Kdm3a* KO mice is not caused by any changes of the estrogen hormone. The data shows that the serum estradiol level is not altered in *Kdm3a* KO mice and the estrogen replacement in the ovariectomized *Kdm3a* KO mice is also unable to rescue the retarded mammary gland growth. Importantly, the ductal outgrowth derived from the transplanted *Kdm3a* KO mammary gland tissue was restricted in both WT and *Kdm3a* KO recipient mice. However, the ductal outgrowth derived from the transplanted WT mammary gland tissue was robust in the WT mammary gland fat pads but only slightly slowed down in the *Kdm3a* KO mammary gland fat pads of recipient mice. These results indicate that the mammary gland epithelial cell-intrinsic Kdm3a plays a major role while the Kdm3a expressed in other types of cells in the recipient environment plays a minor role in promoting mammary gland epithelial proliferation and ductal growth.

As a histone demethylase, Kdm3a can specifically remove methyl residues from H3K9me1 and H3K9me2 and leads to transcriptional activation [3, 5, 6, 10]. Our data demonstrated that KO of *Kdm3a* significantly increased the levels of H3K9me1 and H3K9me2 in the mammary gland epithelial cells of 6- and 8-week-old mice. In contrast, KO of *Kdm3a* did not affect the level of H3K9me3, which is consistent with previous studies showing that Kdm3a mainly demethylates H3K9me1 and H3K9me2 [8]. Overall, these results indicate that Kdm3a plays an important role in supporting the histone code of transcriptional activation for mammary gland ductal growth during perbuty by downregulating both H3K9me1 and H3K9me2.

Kdm3a has been shown to promote several types of cancers including colorectal carcinomas [20], neuroblastoma [21], hepatocellular carcinoma [22] and sarcoma [23, 24]. In this study, we assessed the role of Kdm3a in mammary gland tumorigenesis by comparing the latencies and growth rates of mammary tumors induced by RCAS-PyMT virus in WT and *Kdm3a* KO mice. We found that KO of Kdm3a did not significantly change the tumorigenic latency, but it significantly slowed down the tumor growth rate. In agreement with the notion that Kdm3a can promote breast tumor growth, we found that the proliferation rate of WT tumor cells was much higher than that of *Kdm3a* KO tumor cells. These results indicate that Kdm3a plays an important role in promoting breast tumor cell proliferation and growth, which is consistent with its role in promoting the epithelial cell proliferation and growth in the normal mouse mammary glands during puberty as discussed in a preceding section.

The significantly increased levels of H3K9me1 and H3K9me2 histone codes detected in *Kdm3a* KO mammary epithelial cells suggest that Kdm3a may be involved in the regulation of many genes. It has been reported that Kdm3a can work with androgen receptor to upregulate c-Myc expression in prostate cancer cells [6, 25]. In colon carcinoma cells, hypoxia induces HIF-1 expression and in turn, HIF-1 induces Kdm3a expression. Subsequently, Kdm3a upregulates the expression of VEGF, adrenomedulin and GDF15 to promote cancer cell growth [20]. In order to identify some of the molecular basis responsible for Kdm3a-promoted mammary ductal elongation and tumor growth, we chose to investigate the role of Kdm3a in regulating the expression level of cyclin D1, a cell cycle regulator stimulated by many extracellular growth factors, cytokines and hormones [19, 26, 27]. Our data demonstrated that Kdm3a can directly upregulate cyclin D1 expression to promote mammary gland epithelial cell and tumor cell proliferation. Firstly, we found that Kdm3a expression was positively correlated with cyclin D1 expression and mammary epithelial cell proliferation during puberty. Moreover, KO of *Kdm3a* decreased cyclin D1 expression and epithelial proliferation in the mammary gland during this stage. Secondly, the decreased cyclin D1 expression was associated with the increased H3K9me1 and H3K9me2 levels in Kdm3a KO mammary gland epithelial cells, suggesting that cyclin D1 expression is regulated by Kdm3a-mediated demethylation of H3K9me1 and H3K9me2. Thirdly, we found that KO of *Kdm3a* in the mammary tumor cells decreased cyclin D1 expression and tumor cell proliferation, suggesting that Kdm3a also up regulates cyclin D1 expression and promotes cell proliferation in breast cancer cells. Fourthly, we also demonstrated that Kdm3a was associated with the proximal regions of the cyclin D1 gene promoter. Expression of Kdm3a robustly activated cyclin D1 promoter and upregulated cyclin D1 mRNA expression. Together, these findings indicate that Kdm3a can directly upregulate cyclin D1 expression to promote cell proliferation. However, Kdm3a itself does not bind DNA and it is currently unclear how Kdm3a is recruited to the cyclin D1 promoter.

Intriguingly, Kdm3a was also reported as a tumor suppressor in human germ cell-derived tumors such as seminomas, yolk sac tumors and embryonal carcinomas [17]. In these tumors, Kdm3a is strikingly downregulated, resulting in a loss of its opposing role to the oncogenic H3K9 methyltransferase G9a and an increase in tumor growth. At the molecular level, Kdm3a and G9a drive mutually opposing expression of the antiangiogenic factor genes Robo4, Igfbp4, Notch4, and Tfpi accompanied by changes in H3K9 methylation status [17]. Therefore, Kdm3a can be either a cancer driver or a tumor suppressor, depending on the differential cellular environments of different tumor types. The different role of Kdm3a in different types of cancer may be attributed to the cell type-specific gene expression profiles regulated by Kdm3a. Accordingly, whether Kdm3a can be targeted for cancer treatment should be determined by its tumor-promoting or tumor-suppressing role in the specific cancer type.

In summary, this study revealed that Kdm3a can promote mammary gland epithelial and tumor cell proliferation to facilitate mammary gland ductal elongation and tumor growth through upregulating cyclin D1 expression. These findings indicate that Kdm3a is a key epigenetic regulator in the regulation of mammary gland growth and tumorigenesis.

## MATERIALS AND METHODS

### Mice

*Kdm3a* KO mouse line was initially generated as described previously in a mixed C57BL/6j and 129/SvEv strain background [8] and then backcrossed into C57BL/6j background for at least 6 generations. In *Kdm3a* KO mice, exons 17-25 encoding the demethylase domain of Kdm3a was genetically deleted. The female *Kdm3a* KO and WT mice used for mammary gland analysis in this study were produced from *Kdm3a*-heterozygous breeding pairs. Genotype analysis was performed as described previously [8]. To generate mice for mammary tumorigenesis, *Kdm3a*-heterozygous mice were crossbred with MMTV-TVA transgenic mice to produce female *Kdm3a* WT, heterozygous and KO mice harboring the MMTV-TVA transgene. In MMTV-TVA mice, the TVA viral receptor is specifically expressed in the mouse mammary luminal epithelial cells. RACS virus which contain PyMT expression DNA were injected into mammary gland through mouse nipple to infect TVA positive cells. The expression of PyMT in TVA positive cells could induce rapid tumorigenesis *in vivo* without affecting any other animal or human [28, 29]. The animal protocols were approved by the Institutional Animal Care and Use Committee at Baylor College of Medicine.

### Ovariectomy, hormonal treatment and mammary gland transplantation

Four-week-old *Kdm3a* KO and WT mice were ovariectomized by the dorsal approach as described [30]. Briefly, mouse was anesthetized with isoflurane and mouse hair was shaved at the dorsal-abdominal area. After cleaning the surgical area, a small incision was made parallel to the midline and then the ovary was pulled out from the abdominal cavity. The fallopian tube close to the uterus was ligated with absorbable suture and the ovary was excised with scissors. After the abdominal muscle layer was sealed with absorbable suture, the skin incision was closed by wound clips. These ovariectomized mice were allowed to recover for 2 weeks. A bee’s wax pellet containing 20 µg of estradiol or 20 µg of estradiol and 20 mg of progesterone was implanted under the dorsal skin between the scapulae. After mice were treated with hormonal pellet for 2 weeks, mice were euthanized and their mammary glands were collected for experimental analysis. To determine the systemic influence of global *Kdm3a* KO on the mammary gland development, we performed mammary gland transplantation experiments using mammary gland pieces from *Kdm3a* KO or WT donor mice. The developing mammary gland parenchyma in fourth pair of mammary glands does not extend beyond the lymph node in 3 to 4-week-old weanling mice, surgical removal of developing glands from lymph node using scissors will leave a gland-free fad pad which provide essential microenvironment for the transplanted mammary gland pieces to grow. In our experiment, we surgically removed developing mammary glands from at least 5 *Kdm3a* KO and 5 WT little mates mice at 3 to 4-weeks of age. The incisions on the fat pad were precise and clean. Then mammary glands using for transplantation were isolated from 6-week-old KO and WT donor mice and immersed in sterile PBS in tissue culture dish. Gland tissues around lymph node area which contain ductal structures were cut into 1.0 X1.0 mm pieces with lancet and inserted into pre-cleared mammary glands of 3 to 4-week-old *Kdm3a* KO and WT recipient mice. After transplantation for 4 weeks, mammary glands harboring the transplanted gland tissues were isolated from recipient mice and spread on slides using tweezers, followed by fixing in Carnoy’s solution for morphological analysis. At least 5 mice were included in each group.

### Ductal outgrowth analysis

Whole mount mammary glands from *Kdm3a* KO and WT mice were prepared as described [31]. Briefly, gland tissues were isolated from *Kdm3a* KO and WT mice at the age of 4, 6, 8 and 10 weeks, spread out on microscope slides and placed in Carnoy’s fixative at room temperature for 2-4 hours. After washing the slides with 70% ethanol for 15 mins, followed by rinsing in water for 10 mins, slides were stained by carmine alum staining solution overnight at room temperature. Then mammary glands on slides went through dehydration in gradient ethanol and cleared with xylene for two days. Whole mount images were taken under the dissect microscope.

Mammary gland ductal growth was determined by elongation index, number of terminal end-buds (TEBs) and ductal outgrowth area from binary images as described [32]. In brief, ductal elongation index was obtained by the ratio of the length between lymph node and gland branch leading edge to the total length of the fat pad. The number of TEBs in mammary glands is positive correlated with the cellular proliferation of ductal epithelium and was counted from binary images of KO and WT mammary glands using ImageJ software (National Institute of Health, USA). The ductal outgrowth area was the total area of the gland occupied by the ducts and associated stroma. The area of the mammary gland ductal tree in WT and *Kdm3a* KO mice was measured using ImageJ software.

### Immunohistochemistry (IHC)

IHC was performed as described previously [33, 34]. In brief, mammary gland or tumor paraffin sections were de-paraffinized, re-hydrated and incubated in 10 µM sodium citrate buffer at 95°C for 10 minutes for antigen retrieval. After treatment with 3% H_2_O_2_ for 10 mins to quench the endogenous peroxidase, tissue sections were blocked with 10% normal horse serum for 1 hour at room temperature. The prepared tissue sections were incubated with primary antibodies against Kdm3a (Abcam, dilution 1:1000), Ki67 (Abcam, dilution 1:5000), cyclinD1 (Abcam, dilution 1:200), H3K9me1 (Millipore, dilution 1:500), H3K9me2 (Upstate, dilution 1:500), H3K9me3 (Upstate, dilution 1:500), ERα (Santa Cruz, dilution 1:2000) or progesterone receptor (PR) (Abcam, dilution 1:200) overnight at 4°C. After washing, the tissue sections were further incubated with appropriate biotin-conjugated secondary antibodies. Immunostaining signals were visualized by sequentially incubating with the Avidin-conjugated horseradish peroxidase (HRP) (AK-5200, Vector Laboratories) and its substrate diaminobenzidine (SK-4100, Vector Laboratories). Finally, tissue slides were counter-stained with hematoxylin, sealed in Permount and examined under a Zeiss microscope. For the purpose of semi-quantitative analysis, Ki67-positive and total mammary gland epithelial cells or tumor cells were counted and the percent of Ki67-positive cells was calculated to represent cell proliferation rate. Since the immunostaining signals for H3K9me1, H3K9me2 and H3K9me3 only showed different intensity but no difference in the positive cell numbers, we used a scale of 0-4 scores to estimate their immunoreactivities. For evaluating cyclin D1 IHC, we used Allred scoring system [35], where the immunoreactivity was scored as 0 (negative), 1 (weak), 2 (medium) and 3 (strong) and the percent of positively stained cell number was scored as 0 ( < 0.1%), 1 (0.1∼1%), 2 (1%∼10%), 3 (10∼33%), 4 (33∼66%) and 5 ( > 66%). The final score was the sum of both scores, which gives a 0-8 score range.

### ChIP assay and transfection assay

ChIP assay was performed with MCF-7 cells using Kdm3a antibody as described [29]. DNA samples were prepared from the immunoprecipitated DNA-protein complexes and measured by real-time quantitative PCR (QPCR). For QPCR, three pairs of primers (5’-cgggtcgggattttatgaat / 5’-cgccgggaattaggattact, 5’-gggaccctctcatgtaacca / 5’-ccaccgaaggttcctaattg and 5’-aggaccgactggtcaaggta / 5’- acaacccctgtgcaagtttc) were designed to amplify three DNA fragments in the 5’ none coding regulatory sequence containing the cyclin D1 promoter, which were from -1879 to -1671 bp, -1318 to -1140 bp, and -948 to -687 bp, respectively.

The human cyclin D1 promoter-luciferase reporter was generated in the pGL3 luciferase vector (Promega, Madison, WI). Specifically, a 1643 bp fragment containing the cyclin D1 promoter region was amplified by PCR using a pair of primers (5’-gaatggtaccgatgctctgaggcttggcta and 5’-gatagctagcactcccctgtagtccgtgtg) and inserted into pGL3 vector through the KpnI and NheI cloning sites. Hela cells were cultured to 70% confluence in 24-well plate and then transfected with same amounts of the pGL3-cyclin D1 promoter-luciferase reporter plasmid (200 ng/well) and different amounts of the KDM3A expression vector (0, 100, 200 or 400 ng/well) by using the lipofectamine 2000 reagent. Cells were harvested 48 hours later, and the luciferase activity of each cell lysate was measured by using the Firefly Luciferase Activity kit (Promega, Madison, WI). The relative luciferase activity was obtained by normalizing the luciferase activity to the total protein assayed for each sample.

### Mammary tumor induction and monitoring

The RCAS-PyMT avian virus was packaged in the chicken fibroblast cells and intraductally introduced into the inguinal mammary glands of 8-week-old female *Kdm3a* KO and WT mice as described previously [29]. At least 10 mice were included in each group. Mammary tumor development was examined by palpation once a week after viral introduction. The day when a palpable tumor was detected was recorded for calculating the latency of mammary tumor formation. The length (L) and width (W) of each tumor were measured once a week for a period of 5 weeks as we described previously [34]. Tumor volume was calculated by the formula (L×W×W)/2 as described previously [29, 33].

### Measurement of 17β-estradiol in the mouse serum samples

About 200 µl of blood samples were collected by the periorbital puncture method from *Kdm3a* KO and WT mice at 4, 6, 8 and 10 weeks of age. Blood samples were incubated at 37°C for 1 hour and then kept at 4°C for 4 hours. The serum was isolated after centrifuging at 1,000-2,000 x g for 10 mins at 4°C. The concentrations of 17β-estradiol in the serum samples were measured using the ELISA kits by following the manufacture’s instruction (DSL-4400, Diagnostic Systems, South San Francisco, CA).
